# The Looming Dangers of Explosion in Community Transmissions of COVID-19 in Nigeria

**DOI:** 10.5334/aogh.2990

**Published:** 2020-08-06

**Authors:** Agaptus Nwozor, Charles Okolie, Onjefu Okidu, Segun Oshewolo

**Affiliations:** 1 Department of Political Science and International Relations, Landmark University, Omu Aran, Kwara State, NG; 2 Infectious Disease Research for One Health, Department of Microbiology, Landmark University, Omu-Aran, Kwara State, NG; 3 Mass Communication Programme, Landmark University, Omu Aran, Kwara State, NG

## Abstract

Currently, Nigeria is still at the ascending phase of the COVID-19 curve with no sign of deceleration. Thus, the recent decision by governors of states in northern Nigeria to deport Almajirai (itinerant Islamic school pupils) from their states as part of efforts to contain COVID-19 transmission is likely to have a serious backlash. With hundreds of Almajirai testing positive to COVID-19, and millions of others untested, they constitute ubiquitous nodes of transmission. Their deportation has created multiple emigration channels that constitute prospective feeders to covert community transmission. This viewpoint examines this trend within the context of Nigeria’s current [in]capacity to manage the spread of COVID-19 and concludes that greater risks seem to lie ahead unless the government takes stringent containment measures.

## Introduction

Nigeria’s capacity to manage the spread of COVID-19 in a sustainable manner has been questioned [1,2]. This is so because of a combination of factors, namely the country’s large population, poor healthcare facilities, widespread poverty, illiteracy and lack of infrastructural facilities to aid compliance with preventive measures. These afore-mentioned factors have made it difficult for many Nigerians to observe the routine preventive measures advised by the World Health Organization, such as hand hygiene, social distancing and the use of facemasks [3,4]. So far, Nigeria is still at the ascending phase of the COVID-19 curve with no sign of bending the curve soonest [5].

Nigeria has had various outbreaks of infectious diseases ranging from yellow fever, cholera, monkeypox, Ebola to Lassa fever, among others. None of these infectious diseases has imposed the nature and degree of socio-economic dislocation and hardship as the COVID-19 pandemic. Since the index case of COVID-19 was recorded in Nigeria, the number of confirmed cases has been upswing. The country’s decrepit healthcare system, which has been neglected for decades, has been struggling to be helpful.

The stage for community transmission of COVID-19 in Nigeria was set by the confirmation of the country’s index case on 27 February 2020. The enormity of the COVID-19 challenge in Nigeria manifested when the contact tracing of the movements of the index case showed that he had contacts with 216 persons [6]. This development prompted Nigeria Centre for Disease Control (NCDC) to activate a multi-sectoral emergency operations center (EOC) at Level 3, which is the highest level of response to public health emergencies in the country.

Although the NCDC is making commendable efforts in managing the COVID-19 pandemic in Nigeria, the transmission curve has shown a likelihood of future widespread infection. As of 10th June 2020, the total number of confirmed cases of COVID-19 transmission in Nigeria was 13,873 cases. A further breakdown of the provenance of the confirmed cases showed that while 2 percent (or 289 cases) had travel history, 26 percent (or 3,650 cases) contracted it from such contacts and 72 percent (or 9,934 cases) got it from unknown exposure [5]. The implication of this breakdown is that several nodal points of unknown community transmission exist. Nigeria’s weak capability for effective contact tracing tends to complicate prospects of sustainable containment of community transmission. A further implication is the distressing and gloomy likelihood of sustained community transmission on account of the unknown nature of the mechanisms of transmission.

The recent decision of governors of northern Nigeria to deport Almajirai (itinerant Islamic school pupils) from their states is a major reversal in the efforts to contain the escalation of COVID-19 transmission. Reports have indicated that hundreds of the Almajirai, who managed to be tested for COVID-19 returned positive results [7]. With most of these Almajirai untested, the danger of covert community transmission is more real than imagined. With scientists speculating about new strains and mutations of the coronavirus, greater risks seem to lie ahead in Nigeria [8,9,10]. Mutation can wreak havoc on all aspects of biomedical efforts, including diagnostics and vaccines [11]. Therefore, in order to avoid the likelihood of such a narrative in Nigeria’s COVID-19 management efforts, it is imperative that governments at various levels adopt and invest in relevant modern medical technologies.

## Known and Unknown Trends in the Trajectory of COVID-19 Transmissions

Nigeria’s COVID-19 index case was an Italian man who had flown into Lagos, Nigeria, from Milan, Italy, on 24th February 2020 via Turkish Airlines, which carried 156 passengers according to the flight’s manifest [6]. It was established through the contact tracing of the index case’s movements that he had direct formal and informal contacts with a cumulative total of 216 persons, including persons he met at the airport, the hotel where he lodged, his workplace and 169 passengers who flew into Lagos with him [6]. Although some of the people who had contact with the index case were either quarantined or advised to self-isolate, only one confirmed case was subsequently linked to him [6].

Nigeria activated its highest public health emergency response by opening a multi-sectoral emergency operations center (EOC) led by the NCDC on 28th February 2020. This move helped to provide measures that have contributed to managing the COVID-19 pandemic in the country. This notwithstanding, since 16th March 2020, Nigeria has been recording positive cases on a daily basis [6]. The early recognition by the Nigerian government that effective management of local transmission of COVID-19 would depend on the implementation of stringent measures of detection, prevention, and control led to the adoption of lockdown policy [1]. For instance, Abuja and two states namely, Ogun and Lagos states were locked down, followed by a spate of lockdowns across the country [12].

While the trend in the transmission of the COVID-19 was tracked by the NCDC in Southern Nigeria, the same was not particularly true for the North. This was principally for two related reasons. The first was the inability of healthcare officials in Kano and northern Nigeria to function optimally due to an acute lack of basic equipment, including personal protective equipment (PPE). The second was the closure of the COVID-19 laboratory in Kano as a result of contamination arising from the mishandling of samples by laboratory officials [13]. Both of these factors created serious complications in healthcare delivery in the north and undermined the capacity to trace the trajectory of transmissions [14,15].

The closure of the COVID-19 laboratory in Kano had negative impacts on tests, detection, and contact tracing. The samples collected from states that rely on the laboratory for tests were taken to Abuja [14]. It was in the midst of this laboratory impasse that a strange disease descended on Kano, killing hundreds of people. There was a series of ambivalence, equivocation, and denial about the link of the “mysterious” disease and COVID-19. State authorities dismissed any connection between deaths from the mysterious disease and COVID-19. Other analysts linked the deaths to lack of access to medical attention due to the closure of health facilities in the state [16,13].

The Kano state government claimed that investigations into the immediate and remote causes of the deaths pointed at complications from hypertension, diabetes, yellow fever, Lassa fever, meningitis, and acute malaria and not COVID-19 [16]. The position of the state government was simply speculative as it lacked verifiable scientific evidence. The state relied on what it called “verbal autopsy” for its conclusion. A major inhibition to proper medical autopsies in northern Nigeria is the age-old ethnocultural and religious discouragement of autopsies. However, the medical experts in the Aminu Kano Teaching Hospital, the major referral public hospital in Kano, indicated that before the state’s index case was confirmed on 11 April 2020, the hospital recorded cases with COVID-19-like symptoms [15]. It was reported that several elderly patients who visited the hospital presented symptoms like fever, cough, breathing difficulties, and low oxygen saturation levels [15]. This piece of the evidence contradicted the denials of Kano state government officials about the connection between the mysterious deaths and COVID-19.

The characteristic poor record-keeping of government agencies made it impossible for the reconstruction of the exact number of deaths connected with the so-called mysterious disease. The alarm about the deaths did not come from government officials or health workers but from gravediggers who felt overwhelmed by the sharp spike in the number of graves they had to dig [17]. Anecdotal estimates put the number of the mysterious deaths in Kano in the range of 600 to 680 deaths within a space of fewer than two weeks [18,19]. The same pattern of mysterious deaths manifested in several states across Northern Nigeria, including Yobe, Jigawa, Bauchi, and Taraba states. In other words, these mysterious deaths were not restricted to Kano. Other northern states recorded a substantial number of deaths, thus suggesting a high probability of epidemiological connections [20,17].

Nigeria’s predilection for playing politics with everything, including the COVID-19 pandemic poses a grave threat to the containment of community transmission in a sustainable manner. The governors of northern states where mysterious deaths occurred threw caution to the wind and made great efforts to deny a connection between these deaths and COVID-19. The basis for the denial was the unscientific verbal autopsies they ordered their health officials to carry out. None of the so-called verbal autopsies purportedly carried out appeared to have factored in laboratory test results of the victims prior to their deaths. It flies in the face of logic that northern governors, and even Nigeria’s Presidential Task Force on COVID-19 (PTF), would rely on speculative verbal autopsies instead of scientifically established medical autopsies with a high degree of certainty.

There appeared to be an epidemiological similarity in the pattern of these mysterious deaths in the states they manifested [20,21]. The recent announcement by Nigeria’s Minister of Health that about 979 persons died from the mysterious disease in Kano in April 2020 provides insight about the covert dangers of community transmission [22]. The data that informed the calculation of estimated deaths in Kano were generated from graveyard records in eight municipal local government areas in the state [23]. The PTF, through Nigeria’s Minister of Health, announced a very strong link between Kano’s mysterious deaths and COVID-19 transmission based on circumstantial evidence [23].

## Migratory Trends in the Face of Weak Diagnostic Testing Capacity

The major strategy adopted by the Nigerian governments at all levels, aside from the demand for the observation of personal hygiene and the use of facemasks, was the imposition of lockdowns. Nigeria has not been fantastic with modern diagnostic approaches for infectious diseases [24]. After the initial two-week lockdown announced by the federal government on 30^th^ March 2020, various states began to adopt it to control and contain community transmission [12]. With a continued daily escalation in the number of confirmed cases, an interstate lockdown was imposed on 27th April 2020 based on an agreement between the presidency and Nigeria Governors’ Forum. The idea behind the interstate travel ban in Nigeria was to ensure that cross-border transmissions were controlled.

Several factors have combined to undermine the interstate travel ban, notwithstanding its relevance in containing the spread of COVID-19 pandemic. The first factor is poverty and desperation for survival in the face of the government’s unclear policy thrust on palliative measures. The recent data from Nigeria’s National Bureau of Statistics distressingly showed that 40.1 percent or 82.9 million Nigerians live below its poverty line of N137,430 (the US $381.75) a year [25]. The second factor is the admixture of ignorance and a misplaced sense of invincibility among many Nigerians. This is spawned by the foreign origin and caliber of initial victims of COVID-19 in Nigeria. Thus, the perception is that it is “big men and foreigners’ disease” and, therefore, unlikely to infect them [17]. The third factor that undermined the interstate travel ban is the pervasive corruption that characterizes the Nigerian system. The government officials, including police personnel, assigned to enforce the interstate lockdown saw it as a windfall, an opportunity to make quick money. While the interstate travel ban was in place as a hollow directive, people traveled across states by bribing their way through the checkpoints mounted by different security and other government agencies [26].

A related impetus for the contravention of the interstate travel ban was the deliberate decision of northern governors to dismantle the institution of Almajiri. The Almajiri system is an age-long institution of Islamic tutelage in Northern Nigeria. It is a system of Islamic education practiced in northern Nigeria. Under the Almajiri system, young persons emigrate from their homes and join popular Islamic teachers in the quest for Koranic knowledge. It is estimated that there are between 9 and 9.5 million Almajirai (plural for Almajiri) in northern Nigeria [27,28]. The Almajiri system has been an enduring mechanism for the perpetuation of misery, poverty, illiteracy, drug addiction, criminality, and social disorder in Nigeria. The Almajirai depend on street begging and menial jobs for survival. In the wake of the COVID-19 pandemic, it became obvious that they constituted canon fodders for the rapid spread and community transmission of the virus. Hundreds of Almajirai tested positive to COVID-19, prompting their ban and uncoordinated deportation both to their home states and across Nigeria [29].

## Nodes of Covert Community Transmissions: The Looming Dangers

The ban on the Almajiri system and their subsequent deportation constitute a serious concern for the containment of community transmission of COVID-19. The deportation of the Almajirai was deployed as a strategy to flatten the curve of COVID-19. However, it is most likely to have a reverse effect. While hundreds of them have been forcefully deported from across different states of the federation, some of them have voluntarily decided to flee in all directions. This constitutes a great danger to the management of local transmission as they constitute nodes of community transmission.

Despite the interstate ban on travel, these Almajirai have managed to improvise their movement from northern to southern Nigeria. Most of them intercepted at some state borders hid in cargo trucks hauling food grains, cattle, and other livestock. So many of them intercepted also tested positive to COVID-19 [30]. Some southern Nigerian socio-cultural groups have interpreted the influx of Almajirai to the south from the perspective of conspiracy theory. They accused the northern governors of collaborating and facilitating the southward travel of the Almajirai to upscale the curve in COVID-19 transmission in the south [30].

Although there is no hard fact to sustain the argument of conspiracy theory in this circumstance, there is a clear danger of exacerbation in the transmission curve of COVID-19. The major drivers of community transmission range from poor hygiene, poor medical facilities to the absence of proactive preventive measures. The migrant Almajirai are more or less destitute and therefore lack the resources to live decently. Thus, the danger is that they are most likely going to be active drivers of transmission. As Dawood has pointed out, COVID-19 is a great biological hazard that is highly contagious even during the latency period [31]. The trend in the number of new cases between 1st and 10th June in Nigeria indicates that stringent measures must be put in place to flatten the curve of transmission or at least begin to flatten it.

## Conclusion

Medical experts and epidemiologists have advised that the containment of community transmission will entail the deployment of stringent measures at the macro and micro realms. Macro-level measures include strengthening institutional capacity for detection, prevention, and control, the provision of critical infrastructural facilities to enable heightened surveillance and rapid identification of suspected cases, and provision of facilities for proper medical management [1]. At the micro-level, the measures will include uncompromising adherence to the prescriptions of personal hygiene, social distancing, using facemasks and taking up the responsibility to report symptoms already established as the accompaniments of COVID-19 namely, fever, shortness of breath, cough, respiratory symptoms, and breathing difficulties [32,33].

The looming danger in Nigeria is that the various levels of government have not invested in facilities that will address the harsh living conditions of vulnerable people. The observation of Rashid, Theobald, Ozano, about the challenges of poverty in containing community transmission of COVID-19 in Bangladesh also applies to Nigeria [34]. It may be easy to recommend social distancing, washing of hands with soap and staying at home but the feasibility of making the poor, the marginalized and the vulnerable to adhere to these protocols are remote as the “poor and vulnerable already live on the edge” and may unlikely be in a position to afford them [34]. Thus, the various governments in Nigeria should recognize the necessity of adopting and investing in modern technologies to facilitate speedier data dissemination and analysis in keeping with the principles of precision epidemiology [31].

As a way to avoid the looming danger of a second wave, which may consume more lives than we have witnessed at the moment, governments at all levels in Nigeria must scale-up the continuous capacity of their healthcare sectors. This will make it possible to deal with transmissions in the post-lockdown periods. It is most likely that stigmatization, poor facilities at the isolation centers, continued non-availability of facilities at the health centers, and weak capacity for rapid diagnosis, tracing, and follow-up of potential contacts will compound the challenges of containing the community spread of COVID-19 at the subnational levels. Government efforts in reversing the aforementioned factors are imperative in dealing with COVID-19 pandemic in Nigeria.
